# Quercetin hybrid-hydrogel microparticles modulate gut microbiota and improve memory in an antibiotic-induced dysbiosis rat model

**DOI:** 10.1038/s41598-025-26608-7

**Published:** 2025-11-25

**Authors:** Sherin Joy Parappilly, Deepa Azhchath Vasu, K. A. Athira Krishnan, M. B. Yadukrishnan, Mohind C. Mohan, Meera Pradeep, Ayswaria Deepti, P. A. Aneesa, I. M. Krishnakumar, P. S. Baby Chakrapani

**Affiliations:** 1 Centre for Neuroscience, Department of Biotechnology, Cochin University of Science and Technology, Kochi, Kerala India; 2 Centre of Excellence in Neurodegeneration and Brain Health, Kochi, Kerala India; 3 R&D Centre, Akay Natural Ingredients Kochi, Kochi, Kerala India; 4 Department of Microbiology, Sree Sankara College, Kalady, Kerala India; 5 PSG Institute of Medical Sciences and Research, Coimbatore, India; 6 Department of Biochemistry, Sree Sankara College, Kalady, Kerala India

**Keywords:** Antibiotics, Cognition, Gut-brain axis, Gut dysbiosis, Hydrogel, Quercetin, Neuroscience, Microbiome

## Abstract

Gut microbiota plays a pivotal role in maintaining human health and well-being. Antibiotic-induced dysbiosis disrupts microbial balance and diversity leading to compromised physiological functions and adverse health outcomes. The present study investigates the functional potential of a dietary fiber-based hybrid-hydrogel microparticles formulation of the naturally occurring dietary flavonoid, quercetin (FQ-35), as a prebiotic intervention to restore gut and brain health through modulation of microbiome-gut-brain axis. An antibiotic cocktail was administered to induce gut microbial imbalance, and subsequently assessed the effects of supplementation of FQ-35 (100 mg/kg b.wt, for 14 days) on cognitive performance, gut microbiota, markers of gut and brain integrity, along with histopathological changes. FQ-35 effectively restored gut microbial diversity, demonstrated by a significant enrichment of beneficial taxa such as *Lactobacillus* and *Bacteroides*, alongside the normalization of key gut integrity markers, including ZO-1 and Occludin. Behavioural studies further demonstrated the improvement in memory and restoration of acetylcholinesterase activity, which were impaired by antibiotic cocktail. The intervention also led to the downregulation of pro-inflammatory mediators such as TLR4, TNF-α, and IL-1β. These findings highlight the therapeutic/functional potential of FQ-35 in restoring homeostasis via. the gut-brain axis, indicating its plausible role in the mitigation of dysbiosis.

**Supplementary Information:**

The online version contains supplementary material available at 10.1038/s41598-025-26608-7.

## Introduction

 The gut-brain axis (GBA) represents a bidirectional communication system between the gut and the central nervous system, integrating neural, endocrine, immune, and metabolic pathways. This intricate network is crucial for maintaining homeostasis and regulating essential functions such as mood, cognition, and behavior^1,2^. The gut microbiota, a diverse community of microorganisms, profoundly influences brain functions and cognitive health through the production of short-chain fatty acids (SCFAs), neurotransmitters, and bioactive metabolites, generally known as postbiotics. These metabolites can cross the blood-brain barrier, modulating neuroinflammation, neurogenesis, and neurotransmission^3^. SCFAs such as butyrate, acetate, and propionate help suppress neuroinflammation by modulating microglia and astrocytes toward anti-inflammatory states^4^. They inhibit pro-inflammatory cytokines (IL-1β, IL-6, TNF-α) by blocking pathways like NF-κB and MAPK. SCFAs, especially butyrate, promote neurogenesis by increasing neurotrophic factors like brain derived neurotrophic factors (BDNF), nerve growth factor (NGF), and glial cell line-derived neurotrophic factor (GDNF) through histone deacetylase (HDAC) inhibition, thus promoting neuronal growth, differentiation, and synaptic plasticity, enhancing learning, memory, and mood regulation^5^. Alterations in gut microbiota can significantly disrupt the synthesis and availability of neurotransmitters including serotonin, dopamine, γ-aminobutyric acid (GABA), and norepinephrine^6^. These microbiota-derived neuroactive compounds interact with the enteric nervous system and influence central brain function via the vagus nerve, immune modulation, and endocrine signaling. Such disturbances are implicated in cognitive deficits, including memory impairment, learning difficulties, and increased susceptibility to neurodegenerative diseases^7^. Maintaining a healthy gut microbiome is thus critical for brain health, and targeting the gut microbiota offers promising therapeutic potential for cognitive and neurological disorders.

The widespread use of antibiotics, though indispensable in combating bacterial infections, often indiscriminately eliminates both pathogenic and beneficial bacteria, leading to significant shifts in gut microbiota composition and diversity a condition known as gut dysbiosis^5^. Recent studies demonstrate that antibiotics, used individually or in combinations such as ampicillin, neomycin, vancomycin, meropenem, gentamicin, and metronidazole, can markedly reduce beneficial taxa including *Ruminococcus*,* Bacteroides*,* Lactobacillus*,* Bifidobacterium*, and other SCFA-producing microbes^8,9^. In parallel, this disruption facilitates the overgrowth of pro-inflammatory and opportunistic taxa, such as *Escherichia*,* Shigella*, and toxigenic *Clostridioides difficile*^8,10^. These microbial shifts result in altered gut metabolome profiles, impaired intestinal barrier function, and increased endotoxin leakage, all of which promote systemic and neuroinflammatory responses^8,10,11^. Preclinical models of antibiotic-induced dysbiosis have shown impaired hippocampal neurogenesis, reduced expression of BDNF, and deficits in learning and memory, highlighting the crucial role gut microbiota in maintaining cognitive health^7^. Recent evidence further underscores the association between dysbiosis and cognitive decline, highlighting its role in neuroinflammation and increased vulnerability to neurological diseases^12,13^. This emerging understanding underscores the need for strategies to preserve or restore microbial equilibrium to support cognitive health. Investigating the GBA in the context of antibiotic-induced dysbiosis offers a valuable opportunity to explore interventions such as dietary supplementation. These approaches have the potential to mitigate cognitive impairments associated with dysbiosis by restoring microbial balance, thereby improving neurocognitive outcomes and overall well-being.

Quercetin, a flavonoid present in many vegetables and fruits, is a food additive approved by the United States Food and Drug Administration (USFDA) with potential anti-inflammatory, anti-carcinogenic, neuroprotective, as well as antioxidant properties^14–17^. However, the poor bioavailability owing to its low solubility and rapid metabolism to relatively weak metabolites has been identified as the major limitation for the functional/therapeutic use of quercetin following dietary interventions^18,19^. To overcome this, various strategies have been explored to enhance the oral bioavailability of quercetin, primarily by improving its solubility and gastrointestinal stability. Approaches such as self-nanoemulsifying drug delivery systems, nanostructured lipid carriers, and the use of synthetic emulsifiers have shown promise in preclinical models. However, the translational relevance of these findings is limited, as bioavailability data from animal models often do not accurately predict human outcomes^20,21^. Alternative formulations, including quercetin glycosides, cyclodextrin inclusion complexes, liposomal formulations using lecithin, and hybrid-hydrogel microparticle systems, have been studied for their pharmacokinetic profiles^22^. While some demonstrated improved absorption in humans, limitations such as chemical modification, low drug loading, poor thermodynamic or gastrointestinal stability, liquid state, and reliance on synthetic additives restrict their utility, especially in nutraceutical applications. Various excipients such as cyclodextrins, chitosan, pectin, and lipid carriers have been explored to enhance quercetin’s bioavailability, but limitations related to stability, cost, and biocompatibility remain^22^. In contrast, fenugreek galactomannan-based hybrid hydrogels offer multiple advantages: improved solubility through hydrogel entrapment, prolonged GI retention via mucoadhesion, and a natural, scalable, food-grade profile. These synergistic properties make fenugreek galactomannan an optimal delivery system^20,23^. A fenugreek galactomannan-based natural hydrogel system has demonstrated enhanced bioavailability and functional benefits, underscoring its potential for safe and translational nutraceutical applications^24^. The enhanced bioavailability of the quercetin-fenugreek galactomannan hydrogel is attributed to its self-emulsifying nature, forming stabilized micelles that disperse rapidly under GI conditions, ensuring mucoadhesion, slow transit, and sustained release for improved absorption^25^. In the present study, we hypothesized that a fenugreek galactomannan-based quercetin formulation could mitigate antibiotic-induced gut dysbiosis and associated cognitive impairment by restoring gut barrier integrity, modulating microbiota composition, and attenuating neuroinflammation. To test this, we evaluated its impact on intestinal permeability, microbial diversity, neuroinflammatory markers, and behavioural performance in a rodent model. Given the emerging role of the gut-brain axis in neurodegeneration, such interventions may have broader clinical implications in delaying or managing cognitive decline associated with neurodegenerative diseases.

## Results

### Effect of treatment on memory

Behavioral assessment demonstrated a significant cognitive deficit in the Abx group, while FQ-35 supplementation markedly restored cognitive function, highlighting quercetin’s potential in mitigating dysbiosis-induced cognitive impairment.

#### Novel object recognition test

**Fig. 1:**
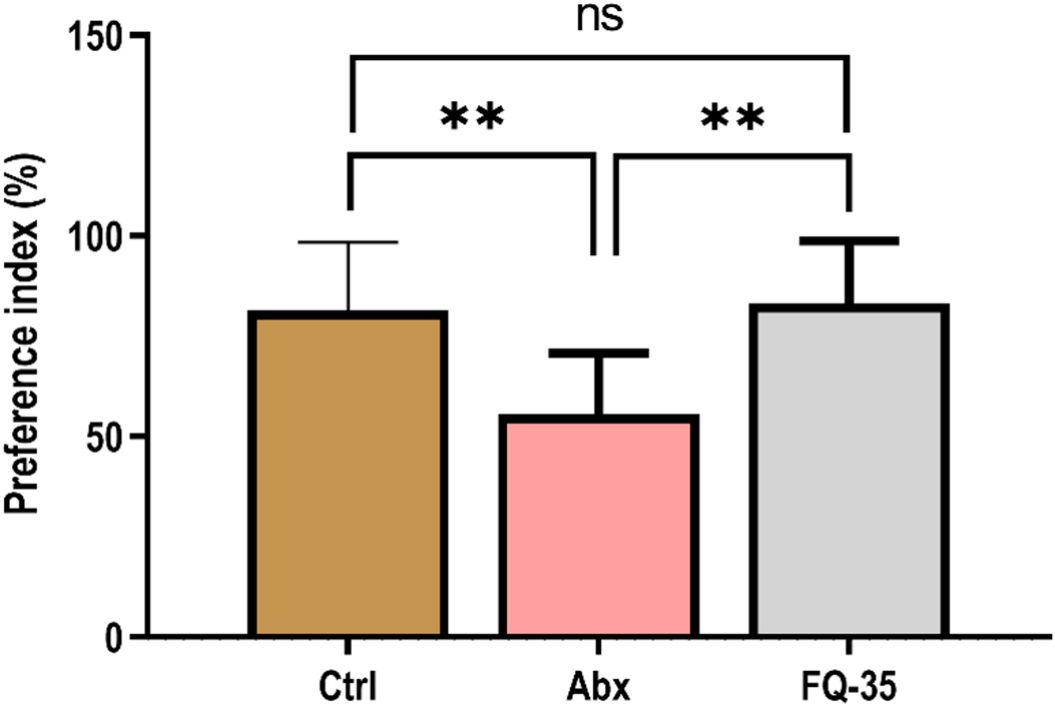
Effect of treatment on recognition memory assessed by NORT. The bar graph shows the preference index in Control (Ctrl), Abx, and FQ-35 groups. **p < 0.001.

To evaluate the impact of antibiotic treatment on learning, and memory, we conducted novel object recognition test (NORT), which measures the time rodents spend exploring a novel versus familiar object to assess memory function. Antibiotic treatment resulted in a significant reduction (*p* = 0.0052) in the preference for novel objects in Abx rodents compared to the Control group (Fig. 1), suggesting memory impairment. Interestingly, FQ-35 supplementation significantly improved (*p* = 0037) the preference index of the FQ-35 group, indicating enhanced spatial recognition memory. Notably, no significant difference was observed between the Control and FQ-35 group (*p* = 0.7910), suggesting that FQ-35 effectively restored memory function to the baseline. The preference index for the novel object in the FQ-35 group was comparable to the Control group, demonstrating FQ-35’s ability to normalize memory function.

#### Y maze

**Fig. 2:**
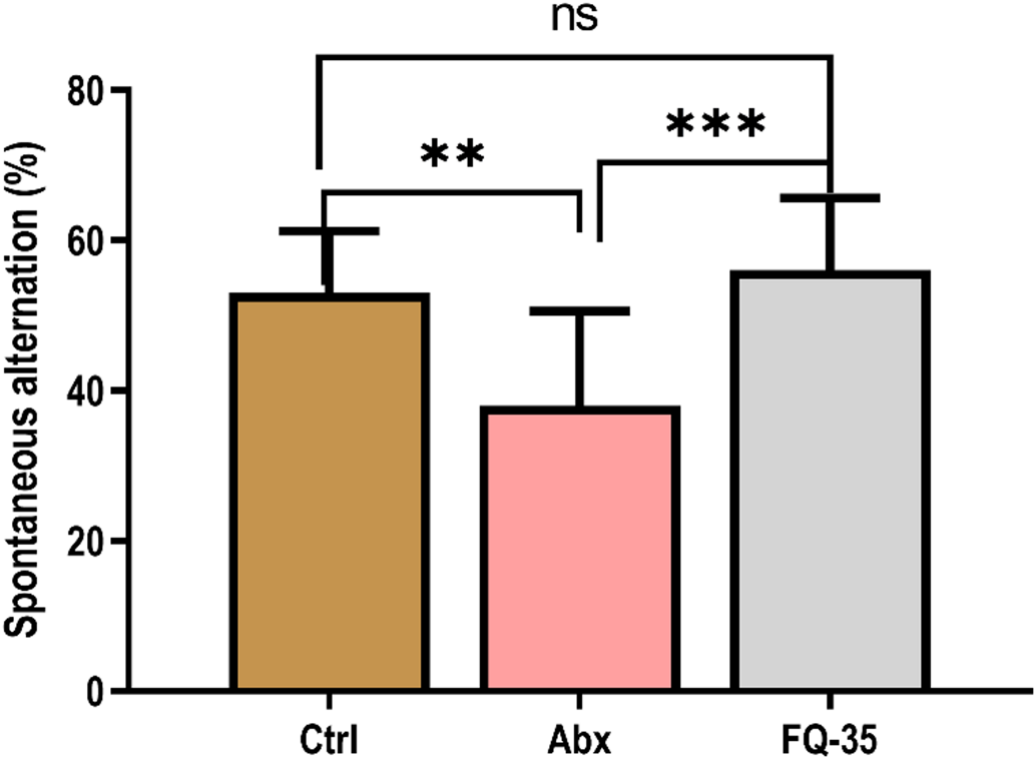
Effect of treatment on spatial memory assessed by Y-Maze test: The bar graph shows the percentage spontaneous alternations in Control (Ctrl), Abx, and FQ-35 groups. **p < 0.01, ***p < 0.001.

In alignment with the NORT assay findings, the Y-maze task demonstrated a significant reduction (*p* = 0.003) in spontaneous alternation, indicative of spatial working memory impairment following antibiotic treatment (Fig. 2). Evidently, FQ-35 supplementation significantly reversed (*p* = 0.0004) this antibiotic-induced deficit, with performance comparable to the Control group (*p* = 0.7546), suggesting the protective role of FQ-35 in preserving or restoring spatial memory function.

## Effect of treatment on gut microbiome profile

The observed improvements in cognition provide a basis for exploring the interplay between gut microbiota and the gut-brain axis. Therefore, microbiome analysis was undertaken to investigate the potential link between gut dysbiosis and cognitive decline, providing insights into the role of the gut-brain axis in mediating protective effect after FQ-35 supplementation.

**Fig. 3:**
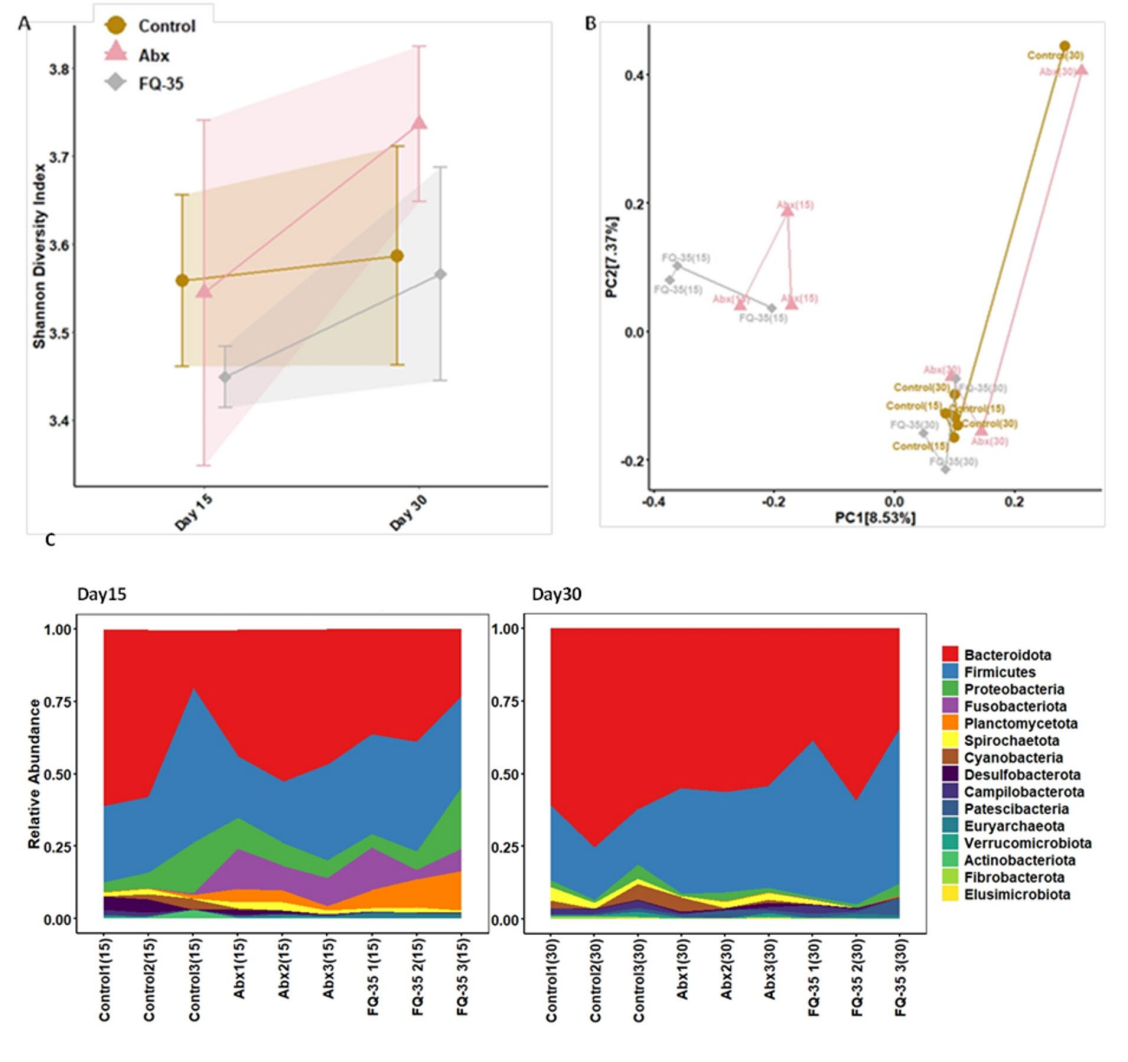
Gut microbiome profile in Control, Abx and FQ-35 groups during post-antibiotic and post-intervention. (**A**) Shannon diversity illustrating a shift in microbial diversity, composition and abundance between the study groups. (**B**) PCoA plot showing Bray-Curtis dissimilarity of microbial composition across the study groups pre and post-treatment with FQ-35. (**C**) Area plot depicting relative abundance of the top fifteen microbial Phylum distribution across Control, Abx, and FQ-35 groups at post-antibiotic and post intervention.

The metagenomic analysis revealed a consistent shift in microbial diversity and composition following antibiotic treatment and subsequent FQ-35 supplementation. The Shannon index indicated a lower diversity and richness of microbial population in the Abx and FQ-35 groups post-antibiotic treatment, suggesting gut microbiota dysbiosis, likely caused by the antibiotics. However, at the end of FQ-35 treatment on Day 30, a significant increase in microbial diversity compared to the Control group was observed (Fig. 3A). Interestingly, the Abx group exhibited a notable shift in diversity, approaching that of the Control and FQ-35 groups at the same time point.

The Principal Coordinates Analysis (PCoA) plot, based on Bray-Curtis dissimilarity showed a clustering pattern revealing distinct differences in gut microbial composition in post-antibiotic treatment and post-intervention groups (Adonis; Permutations = 9999; Group wise pvalue = 0.04; Time-point wise pvalue = 0.001: Pairwise Permanova for Time Point p.adj. = 0.009) (Fig. 3B; Supplementary Table 1). The PCoA plot shows distinct clustering of the three groups on Day 15, with the Control group separated from the Abx and FQ-35 groups (KruskalWallis; p-value = 0.03). The microbial diversity in the Abx and FQ-35 groups was less and compromised following antibiotic treatment, as indicated by the clustering of both groups in the same area on PCoA plot on Day 15. However, by Day 30, the microbial profile of the FQ-35 group showed a shift towards that of the Control group with some overlap, indicating partial recovery of microbial diversity. Furthermore, the FQ-35 group’s microbiota composition remains distinct from the Control group, reflecting ongoing differences due to FQ-35 administration (Pairwise permanova; padj. = 0.01). The relative abundance analysis showed *Bacteroidota* and *Firmicutes* as the dominant phyla across the study groups, with a notable presence of other phyla such as *Proteobacteria*, *Fusobacteria*, *Spirochaetota*, *Cyanobacteria*, *Verrucomicrobiota*, and *Planctomycetota* (Fig. 3C). A drastic shift in the abundant phyla at two time points were also observed. The genus-level distribution followed a similar pattern (Supplementary Fig. 1**)**.

**Fig. 4:**
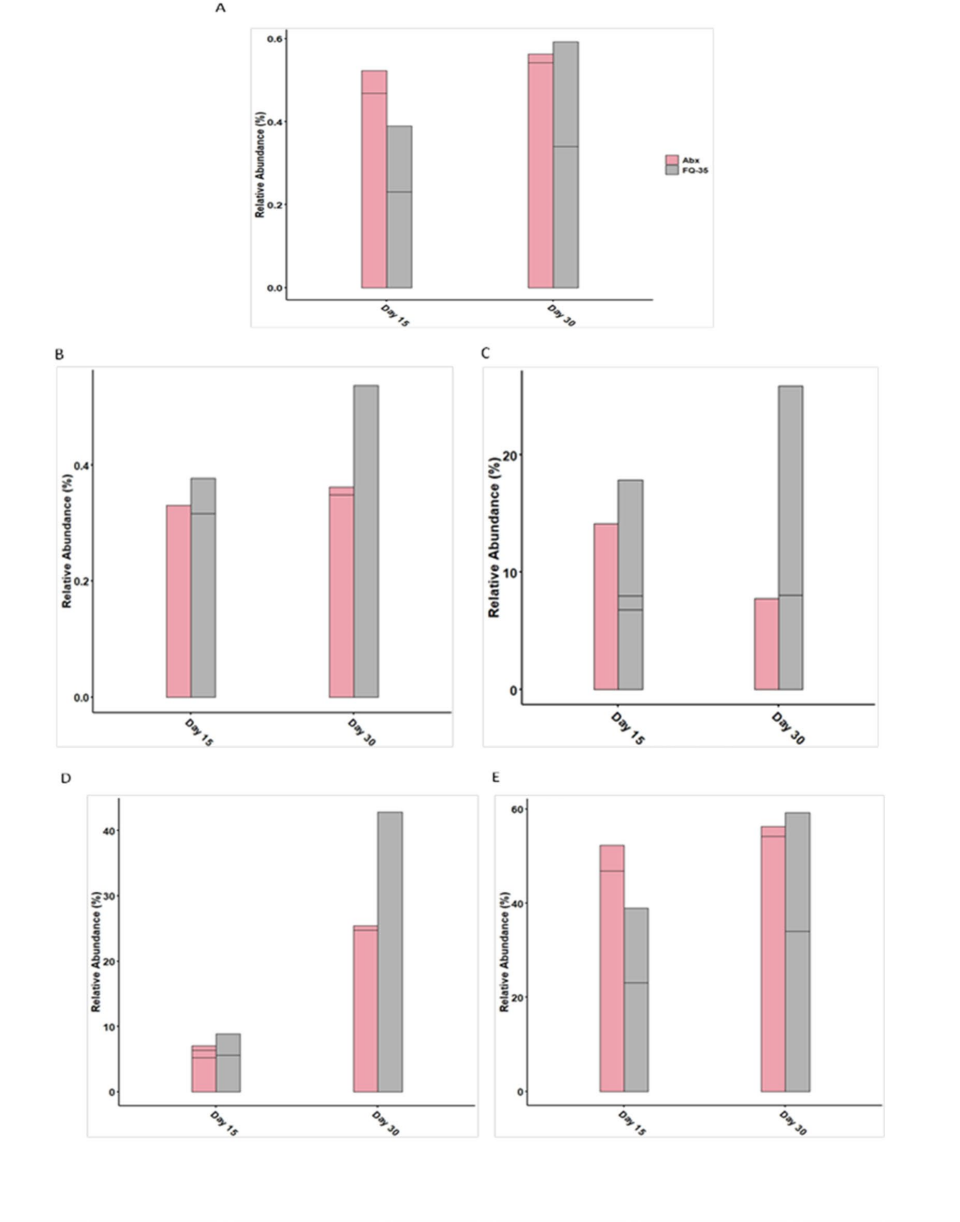
The effect of FQ-35 treatment on the abundant (**A**) *bacteroidota* and (**B**) *firmicutes* and beneficial taxa (**C**) *lactobacillus*, (**D**) *clostridia* and (**E**) *Bacteroidia*. The barplot represents the abundance pattern of significant taxa in the antibiotic induced Abx and FQ-35 groups at two time points (post-antibiotic and post intervention period).

Among the phyla, *Bacteroidota* and *Firmicutes*, exhibited noticeable shifts in abundance following FQ-35 treatment. FQ-35 treatment facilitated an increase in *Bacteroidota* by Day 30, compared to its lower abundance on Day 15 (Fig. 4A) and significantly enhanced the abundance of *Firmicutes* over time compared to the Abx group (Fig. 4B). At the genus level, taxa such as *Lactobacillus*, *Clostridia*, and *Bacteroidia* also demonstrated significant changes in abundance post-antibiotic and post intervention (Fig. 4C-E). An increase in *Lactobacillus* and *Bacteroidia* abundance, was noted in FQ-35 group, particularly after FQ-35 supplementation. Post intervention, FQ-35 group showed a more substantial increase in *Clostridia*.

**Fig. 5:**
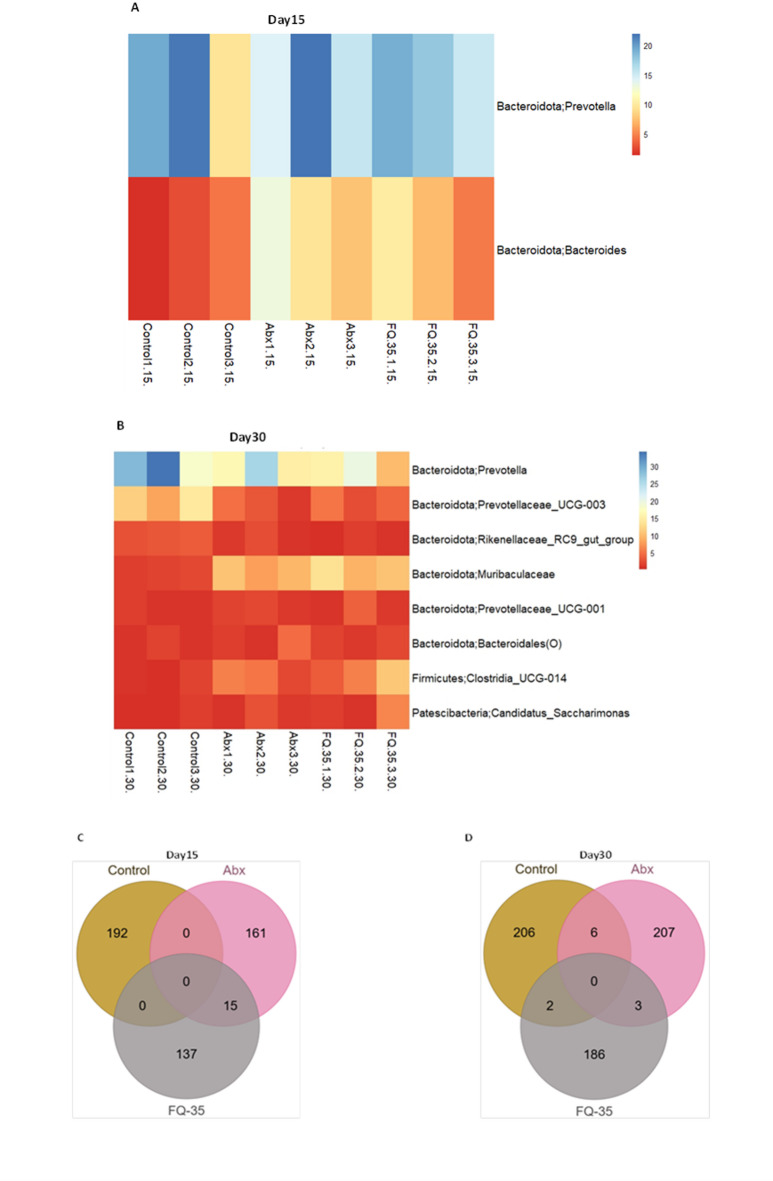
The impact of antibiotic and FQ-35 treatment effect on the core microbiome and the exclusive taxa. The heatmap displays (**A**) Two genera of core microbiome distribution across the groups in the antibiotic dysbiotic gut (**B**) Eight core genera distribution across the groups after the FQ-35 treatment (C-D) Venn diagram showing the number of unique and shared OTUs in different groups in pre-antibiotic treatment (**C**) and post-intervention (**D**).

Core microbiome analysis revealed the consistent presence of *Prevotella* and *Bacteroides* in nearly 90% of samples across all groups post-antibiotic and post intervention (Fig. 5). Antibiotic treatment significantly reduced the diversity of taxa among these genera in both the Abx and FQ-35 groups (Fig. 5A). However, FQ-35 supplementation promoted an increase in microbial diversity, particularly within *Bacteroides* and *Prevotella*, with the core microbiome expanding to eight genera during the recovery phase post-intervention (Fig. 5B). Furthermore, the present study also examined the relative abundance of core microbiota at both time points (Supplementary Fig. 2), and exclusive taxa in each group pre- and post-FQ-35 treatment to further confirm the observed differences in abundance of these taxa across the study groups (Fig. 5C-D; Supplementary **Table 2**). The Control group which consistently exhibited the highest diversity (Shannon index), showed 192 and 206 OTUs on Days 15 and 30, respectively. Post-antibiotic treatment, a notable decrease in OTUs was observed in the Abx (161) and FQ-35 (137) groups. However, FQ-35 administration restored OTUs in the FQ-35 group to 207 by Day 30.

**Fig. 6:**
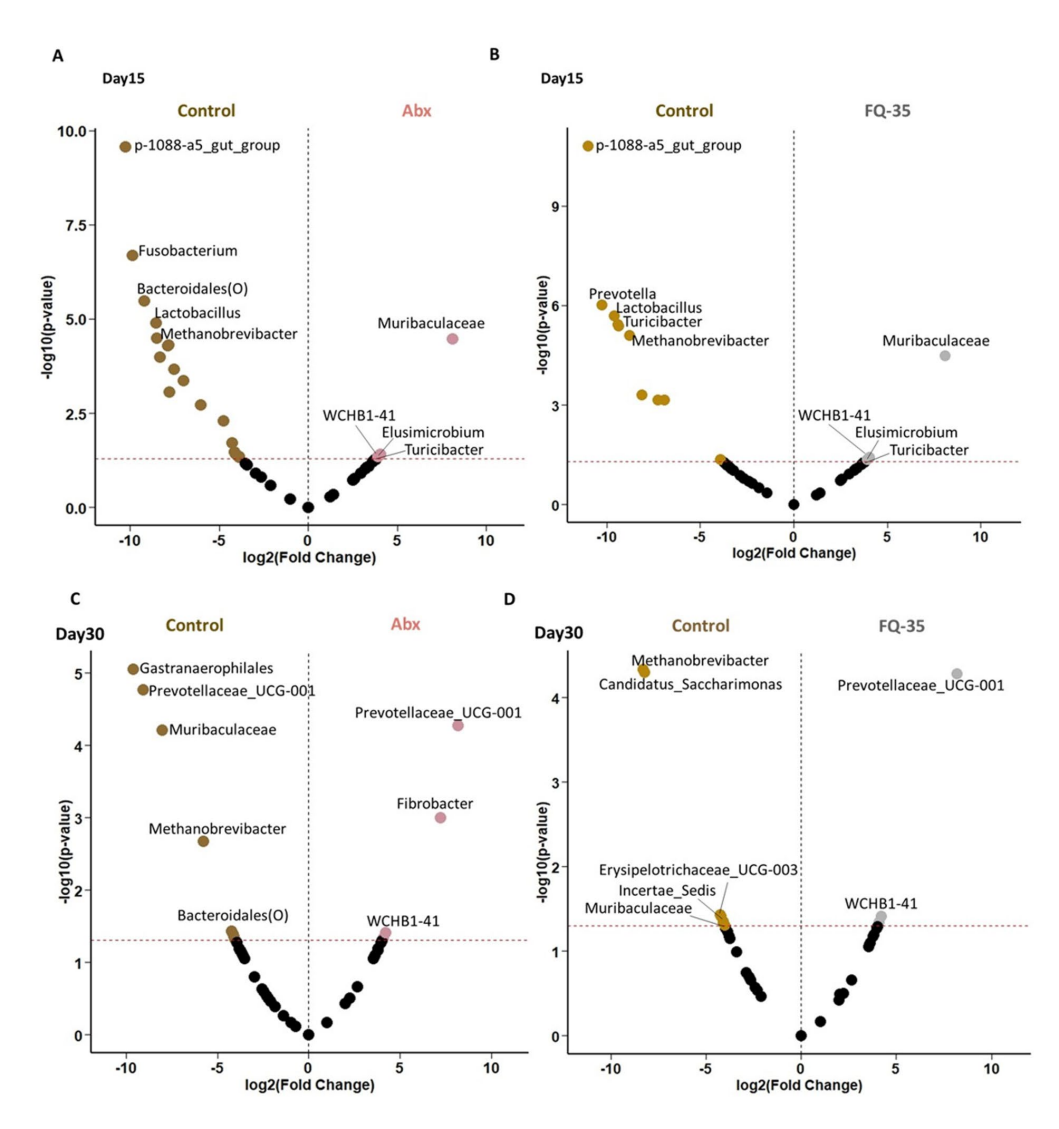
Volcano plots showing differentially abundant bacterial genera identified using DESeq2. Control vs. Abx (**A**) and Control vs. FQ-35 (**B**) comparisons at Day 15 reflect similar microbiota alterations induced by antibiotic treatment prior to intervention. Control vs. Abx (**C**) and Control vs. FQ-35 (**D**) at Day 30 depict post-intervention changes, highlighting the effects of antibiotic withdrawal and FQ-35 supplementation on gut microbial composition. The top five genera with Log2 FC > 1 and p-value < 0.05 is represented in both time points.

Differential analysis using DESeq2 identified taxa that were significantly different in abundance across pairwise comparisons among the Control, Abx, and FQ-35 groups (Supplementary **Table 3**). A comparison between the Control and Abx groups post-antibiotic treatment (Day 15) revealed 23 significantly abundant taxa (*p* < 0.05), with 18 enriched in the Control group and 5 in the Abx group (Fig. 6A). Similarly, analysis between the Control and FQ-35 groups at the same time point showed 10 taxa significantly abundant in the Control group and 5 enriched in the FQ-35 group (Fig. 6B). Notably, microbial composition in the Abx group remained largely consistent even after 14 days of antibiotic withdrawal, with only minor exceptions. Post-intervention, the Control group exhibited significantly higher diversity, with seven taxa enriched, resembling pre-antibiotic conditions, while three taxa were enriched in the Abx group (Fig. 6C). In contrast, post-intervention period, the FQ-35 group displayed microbial diversity and abundance patterns restored to the levels comparable to the Control group, reflecting the recovery of beneficial taxa (Fig. 6D).

By Day 15, the overall microbial diversity remained broadly comparable across groups following antibiotic treatment. Fold-change analysis revealed marked enrichment in specific taxa, notably *Fusobacterium* (*p* = 0.00), *Prevotella* (*p* = 0.00), *Bacteroidales* (*p* = 0.00), and *Selenomonadaceae* (*p* = 0.04) post-antibiotic treatment (Supplementary Fig. 3 A). Interestingly, on Day 30 post-intervention period, seven taxa exhibited significant enrichment in the FQ-35 group compared to Abx group. Among these, *Gastranaerophilales* (*p* = 0.00), *Prevotellaceae_UCG-001* (*p* = 0.00), *Muribaculaceae* (*p* = 0.00), *Methanobrevibacter* (*p* = 0.00), and *Clostridia_UCG-014* (*p* = 0.04) were the top five most abundant taxa (Supplementary Fig. 3B). While, *Methanobrevibacter* (*p* = 0.00), *Candidatus saccharimonas* (*p* = 0.00), *Fibrobacter_intestinalis* (*p* = 0.01), *Erysipelotrichaceae_UCG-003* (*p* = 0.01), and *Incertae_sedis* (*p* = 0.01) were the top 5 abundant in the Abx group after post antibiotic treatment (after 14 days of antibiotic withdrawal).

### Evaluation of the effect of treatment on gut and brain

Investigation on impact of treatment on gut and brain employing histopathological and molecular analyses demonstrated the protective role of quercetin formulation post treatment.

## Intestinal histopathology

**Fig. 7:**
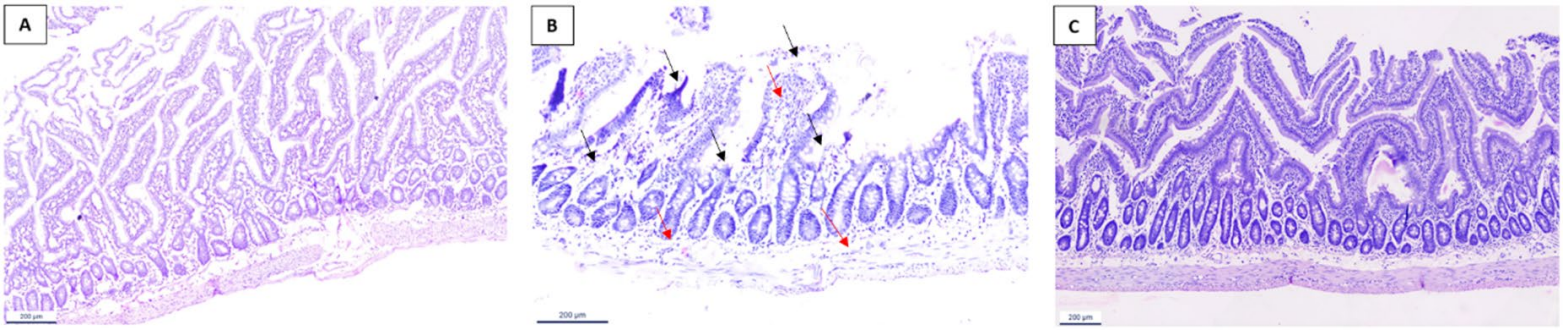
H & E-stained images of intestinal tissue sections of (**A**) Control, (**B**) Abx, and (**C**) FQ-35 groups. The epithelial degeneration and loss of villi and crypts in Abx group is marked with black arrow and inflammatory cell infiltrations marked with red arrows in Abx group.

Histological examination of intestinal sections under light microscopy revealed that antibiotic-treated rodents exhibited a marked (+++) degree of multifocal epithelial degeneration, characterized by extensive loss of villi and crypts (black arrow), along with a moderate (++) infiltration of inflammatory cells within the submucosal and villous regions (red arrow). In contrast, the FQ-35–treated group showed notable histological improvements, supporting its potential role in restoring intestinal mucosal integrity and preserving overall tissue architecture. The Control group displayed normal tissue morphology (Fig. 7A-C).

## Gut integrity and neuroinflammatory markers

**Fig. 8:**
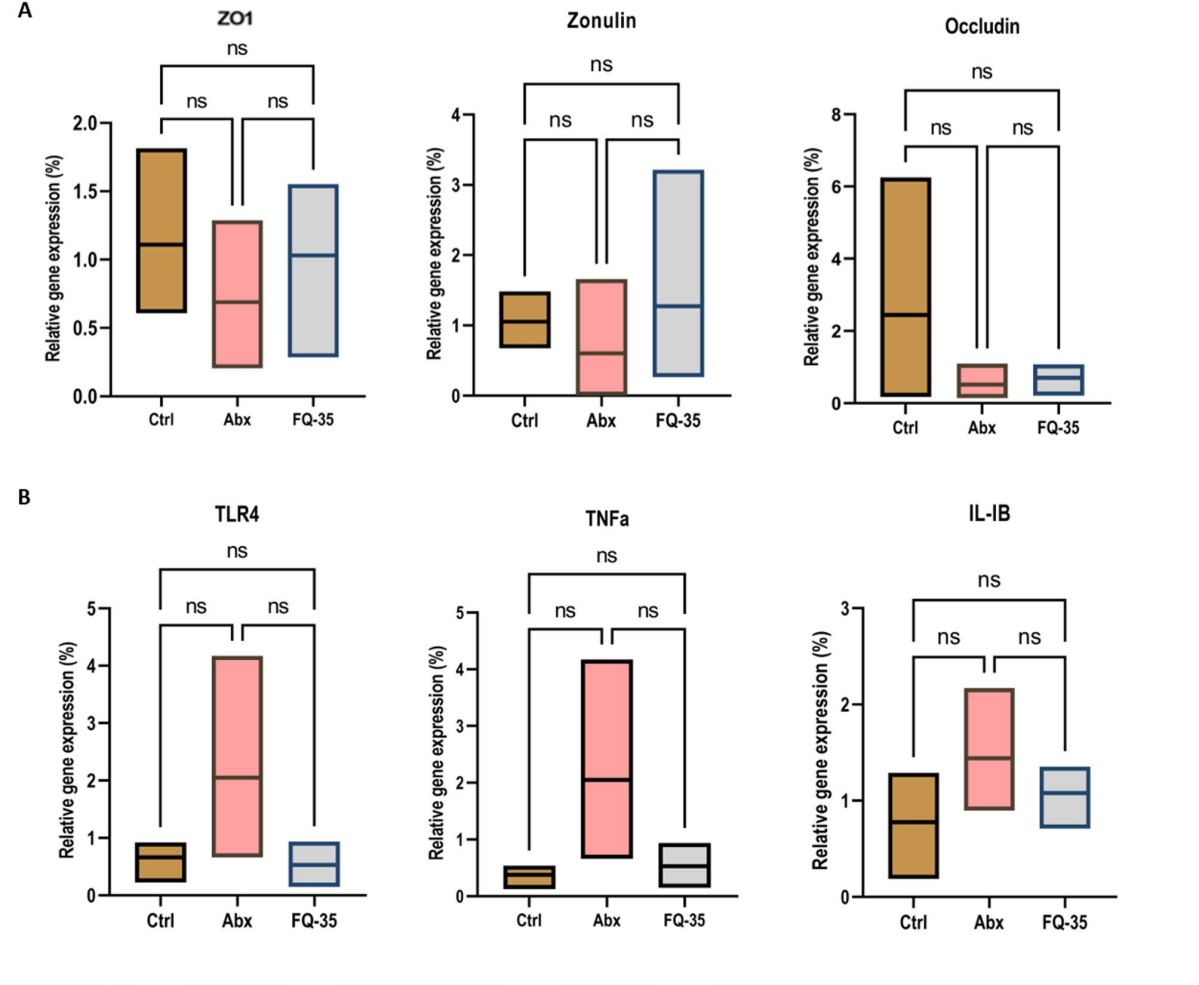
Gene expression profile of gut and brain integrity markers (**A**) The expression profile of gut integrity markers ZO1, Zonulin, and Occludin in Control (Ctrl), Abx and FQ-35 groups. (**B**) Gene expression profile of proinflammatory markers TLR4, TNFa, and IL-1B in the brain tissue across study groups (Control (Ctrl), Abx, and FQ-35).

The quantification of the expression of genes involved in gut integrity (ZO-1, Occludin, and Zonulin) using qRT-PCR showed that the levels of a key gut integrity protein, ZO-1, was low in the Abx group compared to the Control group (Fig. 8A). In contrast, the mean abundance of ZO1 levels was high in the FQ-35 group. Occludin gene expression was reduced, while zonulin levels were elevated in the FQ-35 group compared to the Control group.

Following the evaluation of the intestinal alteration, we also evaluated the antibiotic-mediated changes in the brain to verify the impact of treatment on neural inflammation. qRT-PCR analysis revealed an upregulation of inflammatory markers TLR4, TNF-a, and IL-1B in the Abx group compared to the Control group (Fig. 8B). Notably, FQ-35 supplementation post antibiotic administration effectively reduced the levels of proinflammatory markers associated with antibiotic treatment.

### Acetylcholine esterase

**Fig. 9:**
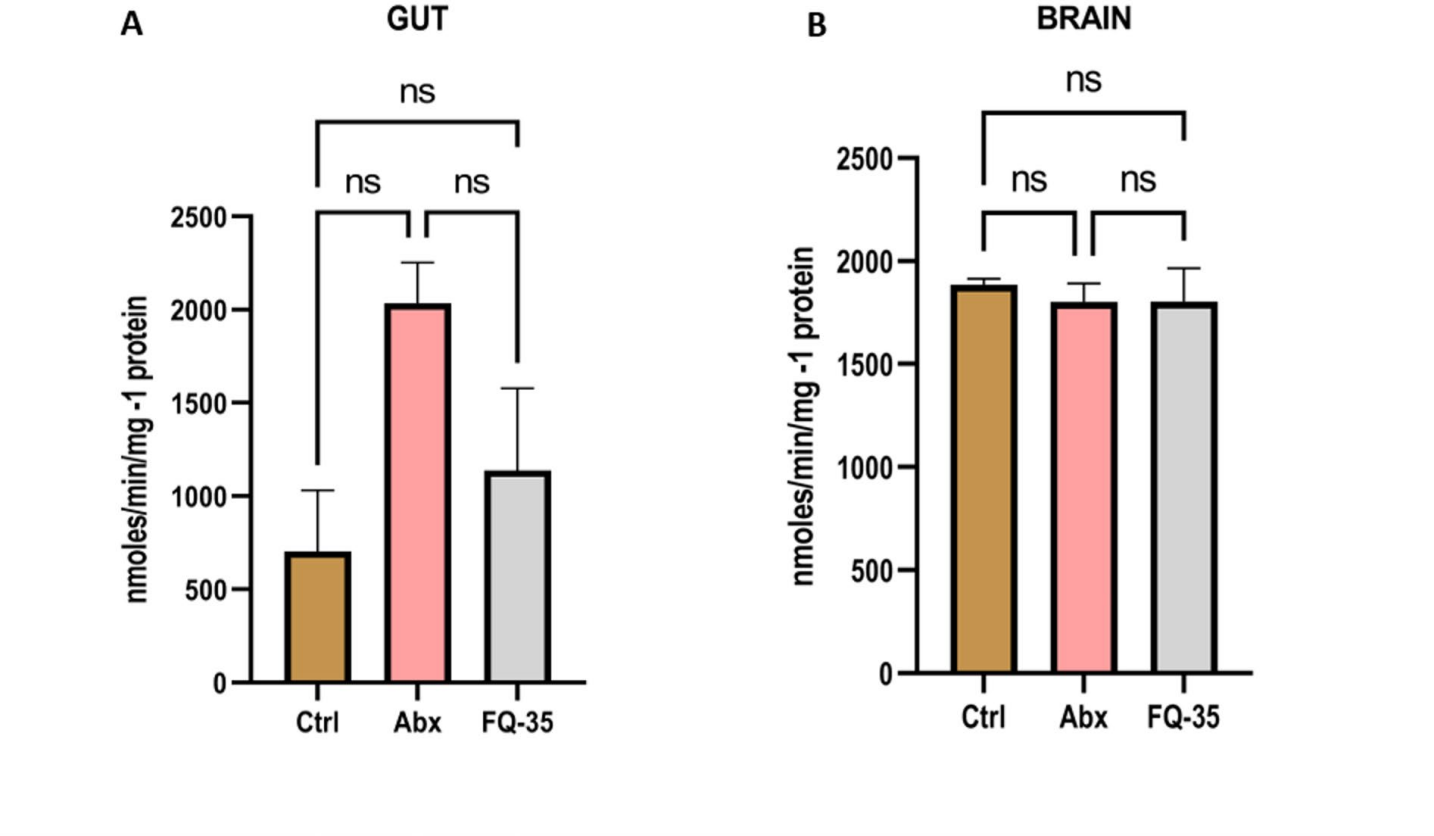
Bar plot showing AchE activity in (**A**) Gut and (**B**) Brain tissues from Control (Ctrl), Abx and FQ-35 groups. One unit is defined as the 1 micromoles of Ach liberated per minutes per mg protein under standards conditions.

The antibiotic treatment resulted in a significant increase in the acetylcholine esterase activity in both the gut and brain, compared to the Control group, indicating reduced levels of circulating acetylcholine (Ach), suggesting impaired gut motility and neuronal activity. However, the FQ-35 supplementation restored AchE activity to a level comparable with Control group demonstrating the efficacy of FQ-35 in normalizing the circulating Ach and AchE activity, ensuring a homeostasis (Fig. 9).

## Discussion

The present study evaluated the efficacy of a natural, food-grade quercetin formulation utilizing fenugreek mucilage hydrogel, (FQ-35), in restoring a healthy gut microbiome in a dysbiotic rodent model. Dysbiosis was induced in Sprague-Dawley (SD) rats using a combination of broad-spectrum antibiotics (ampicillin, neomycin, and vancomycin) chosen for their bactericidal activity against Gram-positive and Gram-negative bacteria^26^. Antibiotic-induced dysbiosis of the gut microbiota can significantly compromise overall health by disrupting the microbial balance and diversity essential for various physiological functions^27–29^. Frequent antibiotic use has been associated with disruption of gut microbiome structure and impairment of cognitive functions^12,30–32^. Therefore, this study examined the potential of FQ-35 to restore a healthy gut microbiome and improve cognitive abilities in the dysbiosis model.

Current scientific understanding suggests that naturally occurring bioactive molecules (phytonutrients) as dietary supplements can modulate the gut microbiota, thereby exerting an influence on the gut-brain axis^33–36^. Quercetin, one of the most abundant flavonoids found in the daily diet, has been shown to positively influence gut health, thus helping brain health^37–40^. In the present study, we employed a hybrid-hydrogel microparticles formulation of quercetin using fenugreek soluble dietary fiber, which has been shown to exhibit improved bioavailability upon oral administration^24^. Previous research on fenugreek soluble dietary fiber have shown its prebiotic potential^41–43^. Therefore, we hypothesized that the hydrogel part being a prebiotic dietary fiber would support beneficial effect on the gut microbiota, in addition to the enhanced bioavailability of the formulation.

Our study observed significant deficit in cognitive function, in response to antibiotic treatment. These findings align with previous research that has linked antibiotic-induced dysbiosis to impairments in cognitive functions, such as recognition and memory^44^. The NORT and Y-maze tests confirmed these deficits, highlighting a reduction in both recognition and spatial memory, likely due to the disruption of gut microbiota. This supports the growing body of evidence that gut health plays a critical role in regulating brain function via GBA. Remarkably, our results demonstrated that supplementation with FQ-35 effectively reversed the cognitive deficits caused by antibiotics. Animals treated with FQ-35 showed significant improvements in both recognition and spatial memory, as evidenced by their performance in the behavioral tests. These findings are consistent with previous studies, which have demonstrated FQ-35’s ability to enhance cognitive function in animal models of neurodegenerative diseases, such as Alzheimer’s disease^45,46^.

To elucidate the relationship between gut microbiota and cognitive function, we analyzed the microbial composition to determine whether the observed behavioural changes were associated with alterations in gut microbiota. Antibiotic treatment induced a marked reduction in microbial diversity and a significant shift in gut microbiota composition, consistent with dysbiosis. These findings align with prior studies demonstrating that antibiotics diminish microbial richness and evenness, leading to a less diverse and potentially unstable gut environment^47–49^. Bray-Curtis dissimilarity analysis revealed a stable microbial composition in the Control group over time, underscoring its resilience. Post-antibiotic treatment, both the Abx and FQ-35 groups displayed distinct separation from the Control group, indicative of similar dysbiosis following antibiotic exposure. Post-intervention, principal coordinate analysis (PCoA) demonstrated a shift in the FQ-35 group toward the Control group, suggesting partial recovery of the gut microbiota. While the clustering of FQ-35 and Control groups remained distinct, the observed shift highlights role of quercetin formulation in promoting beneficial microbial growth^40^. Conversely, the Abx group showed limited recovery following antibiotic withdrawal.

Core and exclusive microbiome analyses further supported these observations. Post-intervention, FQ-35 groups exhibited an increase in exclusive taxa, reflecting recovery attempts from antibiotic-induced disruptions. Core microbiome analysis identified genera such as *Prevotella* and *Bacteroides* as shared across groups, suggesting resilience to antibiotics and FQ-35 intervention^50^. Notably, the FQ-35 group demonstrated additional core taxa post-intervention, highlighting its potential to promote recovery and enhance microbial stability. Differential analysis revealed specific taxa influenced by FQ-35. By Day 30, the FQ-35 group exhibited a more diverse and balanced microbiota, with an increase in key beneficial taxa, such as *Lactobacillus* and *Clostridia*. This contrasts with the Abx group, which showed dysbiosis with overrepresentation of taxa like *Fusobacterium*, reflecting incomplete recovery or persistent microbial disruption^51^. The increased abundance of *Lactobacillus* and *Clostridia* in the FQ-35 group further underscores FQ-35’s role in fostering a healthy microbial community, essential for gut health and overall well-being^52^.

Similar to findings by Shi et al., FQ-35 supplementation enhanced the abundance of beneficial bacteria, including *p-1088-a5_gut_group*, *Bacteroides*, *Lactobacillus*, and *Methanobrevibacter*^40^. The increased abundance of *p-1088-a5_gut_group* (phylum *Planctomycetota*) and *Bacteroides* (phylum *Bacteroidota*), known for their roles in digestion and inflammation reduction, suggests that FQ-35 supports gut health^53,54^. Additionally, the proliferation of *Methanobrevibacter* (phylum *Euryarchaeota*), a methanogenic genus involved in gut fermentation, indicates FQ-35’s potential influence on energy harvest through altered fermentation processes^55^. At baseline, we also observed elevated levels of taxa commonly associated with dysbiosis and impaired gut-brain axis function—namely *Muribaculaceae*, *Elusimicrobium*, and *Turicibacter*—which are linked to conditions such as IBD and type 2 diabetes^56,57^. These taxa were markedly reduced by Day 15 and normalized by Day 30, suggesting progressive microbiota recovery. Concurrently, enrichment of *Prevotellaceae_UCG-001* and *WCHB1-41* were noted by Day 30, both associated with mucin degradation and SCFA production, indicating a shift toward a more resilient and gut-supportive microbial profile potentially beneficial for cognitive function^58^. Consistent with earlier studies quercetin-like polyphenol-based dietary interventions, such as FQ-35, have been shown to reshape gut microbiota by enriching SCFA-producing taxa including *Lactobacillus*, *Clostridia*, *Bacteroides*, and *Prevotellaceae*, and modulate metabolic pathways implicated in brain disorders^52,59,60^. Notably, by Day 30, the FQ-35 group exhibited significant enrichment of several beneficial taxa, including *Gastranaerophilales*,* Ruminococcaceae*,* along with Prevotellaceae_UCG-001*,* Muribaculaceae*,* Methanobrevibacter*,* Bacteroidales* (order), and *Clostridia*_UCG-014. *Gastranaerophilales* has been associated with the restoration of impaired gut function, and *Ruminococcaceae*, a butyrate-producing bacterium which has been linked to improvements in cognitive function^61,62^. These compositional and metabolic shifts may enhance gut-brain communication by strengthening intestinal barrier integrity, reducing systemic inflammation, and influencing neurotransmitter regulation^63^. This, in turn, can affect cognitive processes through mechanisms involving the vagus nerve and microbial metabolites such as GABA and serotonin precursors, potentially contributing to the observed behavioural improvements^63^. Together, our findings demonstrate that FQ-35 not only restores microbial diversity following antibiotic-induced dysbiosis but also promotes the enrichment of beneficial taxa. This comprehensive modulation of the gut ecosystem highlights the therapeutic potential of FQ-35 in restoring gut health and stabilizing microbial communities, particularly in the context of antibiotic-associated disruptions.

Histopathological analysis of the gut revealed significant structural impacts of antibiotic treatment on the intestinal mucosa, corroborating behavioral and microbiota findings. Consistent with prior research linking antibiotics to increased intestinal permeability and inflammation^64,65^, our analysis showed marked tissue alterations in the antibiotic-treated group, including inflammatory cell infiltration, epithelial denudation, and disruption of intestinal crypts. In contrast, FQ-35 supplementation mitigated these effects, with the treated group exhibiting minimal epithelial degeneration, reduced crypt disruption, and lower inflammatory cell infiltration. These observations may indicate a potential role of FQ-35 in aiding intestinal morphological recovery, complementing its effects on microbiota diversity. This restoration of gut integrity underscores its potential in supporting gut-brain axis function and overall health.

Building on the histopathological findings, we further examined the molecular mechanisms underlying gut structural changes by analyzing the expression of key gut integrity markers, including ZO1, Occludin, and Zonulin. While the observations were not statistically significant, the trends observed aligned with the hypothesis. ZO1, a crucial protein for maintaining gut barrier function, was notably reduced in the antibiotic group compared to the Control, consistent with histological evidence of compromised gut integrity. FQ-35 supplementation showed a trend toward restoring ZO1 and Occludin expression, which may indicate a supportive effect on tight junction integrity. However, these differences did not reach statistical significance; the interpretation remains inconclusive, and further studies are needed to establish a definitive link. Zonulin expression showed a slight upward trend in the FQ-35 group, which did not parallel the histological improvements or the restoring effect observed with the expression of other tight junction proteins. This inconsistency in gut integrity markers, along with the concomitant improvement in histological architecture, likely reflects the multifactorial regulation of gut permeability. Previous studies have also reported elevated expression of tight junction genes following probiotic supplementation^66–68^. Given these complexities, the observed trend necessitates further studies incorporating additional gut integrity markers, and larger sample sizes are warranted to achieve a more comprehensive assessment of barrier modulation by FQ-35 supplementation.

In addition to assessing gut integrity, we evaluated the impact of FQ-35 supplementation on the inflammatory response induced by antibiotics, focusing on neuroinflammation. Given the link between gut dysbiosis and brain inflammation via the gut-brain axis, we examined key pro-inflammatory markers, including TLR4, TNF-a, and IL-1B. The antibiotic-treated group showed increased expression of these markers, reflecting elevated neuroinflammation consistent with cognitive decline^69^. Notably, FQ-35 supplementation led to a significant downregulation of IL-1B, with levels normalizing to those observed in the Control group. This downregulation trend extended to all three inflammatory markers, highlighting the anti-inflammatory effects of FQ-35. These results suggest that FQ-35 mitigates the neuroinflammatory response linked to antibiotic-induced dysbiosis^70,71^, which aligns with the cognitive improvements observed, demonstrating the potential of FQ-35 to support both gut and brain health by reducing inflammation and promoting homeostasis.

**Fig. 10:**
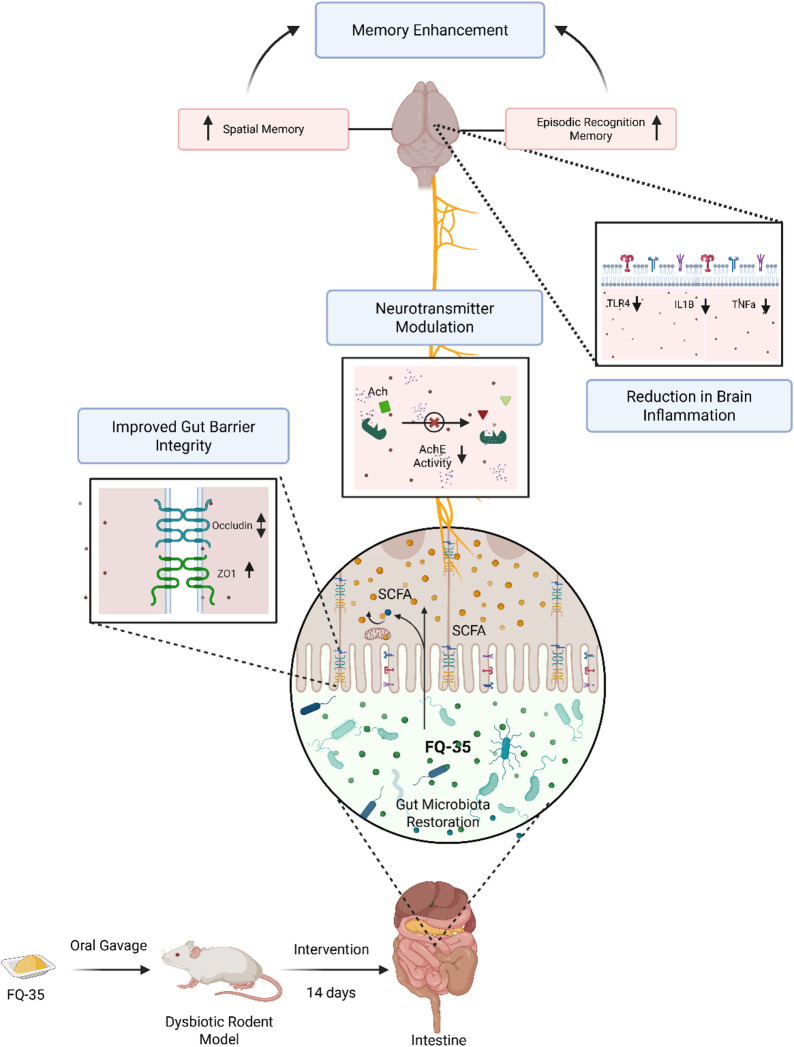
Proposed mechanism illustrating the role of FQ-35 supplementation in restoring gut microbiota composition, enhancing gut integrity, and improving memory function in an antibiotic-induced rodent dysbiosis model.

The evaluation of FQ-35 supplementation revealed increased AchE activity in the gut, suggesting restoration of cholinergic homeostasis. Antibiotic treatment elevated AchE activity, indicating reduced acetylcholine (Ach) levels and impaired gut motility, consistent with dysbiosis and cognitive decline^72^. FQ-35 supplementation normalized AchE activity in the gut, supporting its role in restoring cholinergic balance. Since acetylcholine protects gut health by preventing tissue damage and bacterial imbalances^73^, FQ-35’s ability to downregulate AchE and restore acetylcholine levels highlights its potential to maintain neurotransmitter balance and gut function. These findings align with previous studies^74,75^. Although FQ-35 also affected AchE activity in the brain, the impact was less pronounced, suggesting that further investigation is needed to fully understand its effects on brain function. Based on our findings, we propose a mechanism by which FQ-35 may enhance cognitive function through modulation of the gut-brain axis (Fig. 10). The prebiotic galactomannan and flavonoid quercetin in FQ-35 appear to restore gut microbial balance by enriching beneficial taxa. This microbial shift may increase microbiota-derived metabolites, including neurotransmitters like acetylcholine and SCFAs, which serve as key mediators of gut-brain signalling. These molecules are hypothesized to strengthen gut barrier integrity via upregulation of tight junction proteins and to attenuate neuroinflammation through vagal and systemic immune pathways. Together, these effects may support hippocampal function and underlie the observed improvements in spatial and recognition memory, suggesting a potential link between FQ-35-mediated gut restoration and cognitive outcomes.

This study contributes to the growing field of gut-brain axis research, underscoring the potential of microbiota-targeted dietary strategies. With rising interest in personalized nutrition and the microbiome’s role in neurodevelopmental and neurodegenerative disorders, future studies could explore customizing such formulations based on individual microbiota profiles and extending their application to broader spectrum of gut-brain axis–related disorders.

## Conclusion

FQ-35, a natural food-grade hybrid-hydrogel microparticles formulation of quercetin, supplementation for 14 days at 100 mg/kg b.wt. demonstrated significant therapeutic potential to both gut and brain functions by targeting the gut-brain axis. The treatment restored gut microbial diversity, enriching beneficial taxa that produce SCFAs essential for gut and brain homeostasis. These microbial metabolites enhanced gut barrier integrity by upregulating tight junction proteins like ZO-1, and normalizing Occludin expression. Improvement in gut integrity was further evident from the reduced levels of systemic and local inflammation markers (TLR4, TNF-α, and IL-1β) observed in the FQ-35 group. Furthermore, FQ-35 was found to preserve acetylcholine levels, a key neurotransmitter for cognition, by downregulating AchE activity. Together, these effects may protect neuronal health, and enhance synaptic plasticity through modulation of the gut-brain axis. Collectively, our findings suggest that FQ-35 may hold promise as a nutraceutical or dietary supplement to promote gut-brain homeostasis, and mitigate the adverse effects of antibiotic treatment. Future human intervention studies are highly recommended.

## Materials and methods

### Preparation of FQ-35

An authentic food-grade sample of the hybrid-hydrogel microparticle formulation FQ-35 was provided as a gift by Akay Bioactives, Akay Natural Ingredients Private Limited, Kochi, India. The formulation was produced under Good Manufacturing Practices using the patented FenuMat^®^ technology, which utilizes a fenugreek galactomannan hydrogel scaffold to enhance the solubility and stability of bioactives. FQ-35 was prepared by the uniform impregnation of quercetin micelles, generated using lecithin into the fenugreek hydrogel matrix through high-shear homogenization. The hydrogel was subsequently dehydrated under vacuum to yield an amorphous powder form. High-performance liquid chromatography (HPLC) analysis confirmed that the formulation contained 36.3% total quercetin along with 5% sunflower oil, 20% Lecithin and the remaining portion composed of fenugreek galactomannan. Further details on the formulation process and physicochemical characterization, such as FTIR, SEM, TEM, and particle size analysis (DLS), are available in the original publication^24^.

### Ethics statement

All animal experiments were approved by the Institutional Animal Ethical Committee (IAEC), Cochin University of Science and Technology, India (License No: 328/GO/Re/S/01/CPCSEA/22). All animal experiments were conducted in accordance with the Animal Research: Reporting of In Vivo Experiments (ARRIVE) guidelines, the regulations of the Committee for the Purpose of Control and Supervision of Experiments on Animals (CPCSEA), Government of India, and the International Guiding Principles for Biomedical Research Involving Animals issued by the Council for International Organizations of Medical Sciences (CIOMS). Additional safety and animal welfare measures were adopted as previously described^76–78^.

### Animals

The study was conducted using 8–10-week-old male SD rats purchased from Invivo Biosciences, Bangalore, India. The animals were housed in ventilated cages under strictly controlled conditions, maintaining a temperature of 21 ± 1 °C and a 12-hour light-dark cycle. They had free access to standard chow and water. After a one-week acclimation period, 36 rats were randomly divided into three groups: Control (Ctrl), Antibiotic (Abx), and Quercetin (FQ-35), with 12 animals in each group.

### Treatment

**Fig. 11:**
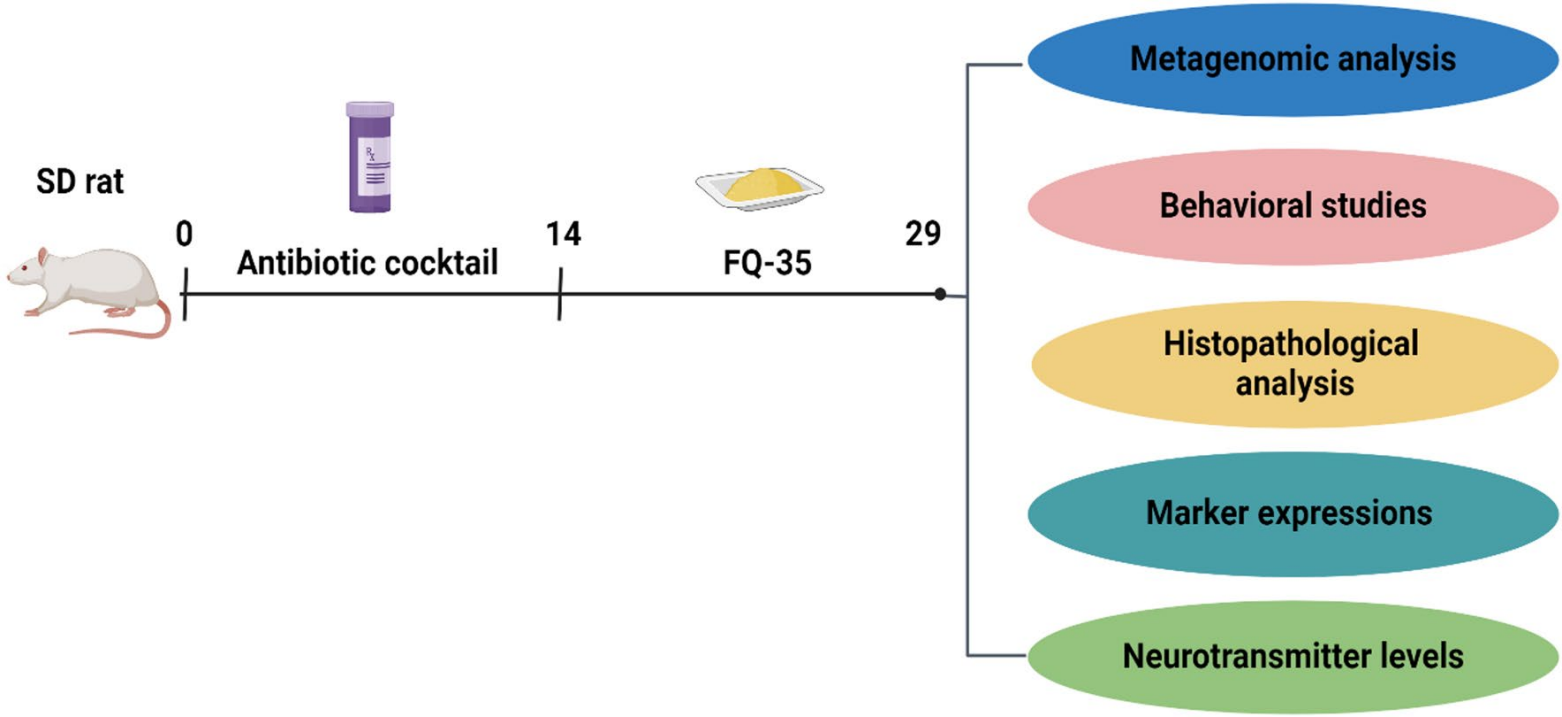
Experimental design illustrating antibiotic and FQ-35 treatment strategies along with the analytical approaches employed in the present study (Created in BioRender. Chakrapani P S, D. (2024) BioRender.com/p10e847).

To induce gut dysbiosis, an antibiotic cocktail consisting of vancomycin, ampicillin, and neomycin was administered orally at a dose of 7 mg/day per animal in both the Abx and FQ-35 groups for 14 days, following the protocol by Zhan et al.^79^. Subsequently, the FQ-35 group received a quercetin formulation (FQ-35, dissolved in water) via oral gavage at a dose of 100 mg/kg b.wt for an additional 14 days and the dose was selected based on previous studies^52,80^. The Control group received an equivalent volume of normal saline (0.85%) for 14 days’ post-antibiotic treatment. Neurobehavioral assessment tests were conducted on Day 20 during the light cycle (9 am – 4 pm IST). Fecal samples were collected from a random subset of three rats per group on Day 15 (post-antibiotic treatment) and on Day 30 (post-intervention) for microbiome analysis. On day 30, all animals were sacrificed under mild anesthesia using isoflurane, and tissues were collected and stored at −80°C for histopathological and molecular analyses, enabling a comprehensive evaluation of gut integrity markers and microbiome composition. An overview of the experimental design is presented in Fig. 11.

### Behavioral studies

To evaluate the therapeutical potential of quercetin formulation, we first investigated the impact of intervention on cognitive function in the dysbiosis model.

### Assessment of memory

The effect of supplementation of FQ-35 on the cognitive changes associated with antibiotic treatment was evaluated by a set of behaviour studies. Behavioural analysis was conducted on the 20th day of treatment using ANY-maze software in a specially designed behavioural study room with minimal disturbances. To minimize stress, animals were acclimated to the test room for 1–2 h before the experiment on each day and handled with utmost care. The recognition memory and the spatial memory of the animals were assessed using NORT and Y-maze apparatus, respectively.

### Novel object recognition test

The NORT was used for the investigation of memory alterations that depends on the intuitive exploratory behaviour without external motivation. On the initial day of the experiment, the rats were habituated in an open rectangular box made of plastic (65 × 45 × 65 cm). Next day, the animals were allowed to familiarize with alike objects (A + A). On the third day (test day), the animals were allowed to explore two unlike objects (A + B). After each trial the box was wiped with 70% ethanol to avoid the odour trails. Cognitive function was expressed as Preference Index (PI), the ratio of the time duration to explore novel objects to the time duration to explore both the objects on the test day. The preference index was calculated using the formula;

$$\:Preference\:Index\:\left(PI\right)=\left[\frac{Time\:Spent\:with\:Novel\:Object}{Time\:Spent\:with\:Novel\:Object+Time\:Spent\:with\:Familiar\:Object}\right]*100$$

A PI > 50% indicates inclination to new object and PI < 50% indicates an inclination to a familiar object. PI of 50% indicates the rodents having equal preferences for both the objects^81^.

### Y-Maze

The Y-maze spontaneous alternation test was utilized to evaluate spatial working memory and exploratory behavior in mice. The apparatus comprised three arms of equal dimensions (labeled A, B, and C) positioned at 120° angles from each other. Each mouse was gently introduced into the distal end of Arm A, oriented toward the center of the maze, and allowed to explore freely for 8 min. The sequence and number of arm entries were recorded throughout the session. Spontaneous alternation behavior, defined as consecutive entries into three different arms without repetition, was quantified to assess cognitive performance. The percentage of spontaneous alternation was calculated using the formula:

$$\:\%\:Spontaneous\:Alteration=\:\left[\frac{Number\:of\:Alterations}{Total\:Number\:of\:Entries-2}\right]*100\:$$

After each trial, the maze was wiped with 70% ethanol solution to prevent any olfactory recognition^76^.

#### Metagenomic analysis of gut microbiome composition 

To investigate the impact of antibiotics on the gut health and further to analyze the effect of FQ-35 supplementation on antibiotic-induced dysbiosis, we performed metagenomic analysis of rat droppings collected at two time points: on Day 15 and Day 30 of treatment, representing the periods post-antibiotic treatment and post-intervention. Fecal samples from each group (Control, Abx, and FQ-35) were collected in sterile containers on Day 15, and Day 30. The samples were stored at −80 °C until further analysis. DNA was extracted using the QIAamp Fast DNA Stool Mini Kit (Qiagen, Hilden, Germany) and assessed for purity. An amplicon library targeting the 16 S rRNA V3-V4 region was prepared using the Nextera XT Index Kit (Illumina Inc, California, USA). The prepared libraries were sequenced on the Illumina MiSeq platform.

The microbiome analysis was carried out using QIIME2 plugins, R, and online metagenomic analysis tools. This encompassed a range of specific statistical analyses, computations for taxonomic annotations, assessments of diversity, and differential analyses. To visually represent the findings, a variety of graphical representations, including bar plots, box plots, and, graphs were generated using R within the RStudio interface (version 6.1.524). To facilitate these analyses, a suite of essential R packages was utilized, comprising *Tidyverse* (version 2.0.0), *dplyr* (version 1.1.4), *plyr* (version 1.8.8), *vegan* (version 2.6–6.1), *DESeq2* (version 1.40.2), *truncnorm* (version 1.0–9), *tibble* (version 3.2.1), *scales* (version 1.3.0), *phyloseq* (version 1.44.0), *tidyr* (version 1.3.1), *effsize* (version 0.8.1), *permute* (version 0.9–7.9), *ggsignif* (version 0.6.4), *stats* (version 4.3.1), *broom* (version 1.0.6), *devtools* (version 2.4.5), *ggrepel* (version 0.9.5), *pheatmap* (version 1.0.12) and *ggplot2* (version 3.5.1). These packages facilitated the comprehensive statistical analysis of the data, allowing for the exploration of taxonomic compositions, diversity metrics, differential abundance testing, and visualization of results.

Alpha diversity was assessed using the Shannon index, and beta diversity was evaluated using the Bray-Curtis analysis to determine within- and between-sample diversity across groups. PCoA plots based on Bray-Curtis dissimilarity were generated using the vegan R package, with PERMANOVA (9999 permutations) employed to assess significant beta diversity differences and pairwise PERMANOVA tests (*pairwiseAdonis* package) to identify specific groups driving these differences. The core microbiome was identified using a threshold of 0.01% relative abundance, and taxa present in at least 90% of the samples within the study groups were included in downstream analysis. A heat map was generated to visualize the core microbiome across the study groups. To explore the unique taxa exclusive to each group, we analyzed the relative abundance of rare taxa across the study groups, aiming to identify specific microbial shifts that occurred before and after intervention. All samples from each group at the two time points were independently analysed and subjected to filtering to identify the shared taxa among Control, Abx, and FQ-35 groups and taxa exclusive to each group in order to detect any changes in microbiota following treatment. A Venn diagram was created to represent the number of exclusive taxa and the shared taxa counts before and after treatment, using an online Venn diagram tool (https://molbiotools.com/listcompare/).

Differentially abundant taxa were identified using DESeq2 in R, applying size factor normalization and modelling data with a negative binomial distribution. The taxa with low counts (minimum count threshold of < 10 counts) across samples are removed to reduce noise. Multiple testing correction is performed to adjust for multiple comparisons to Control the false discovery rate (FDR) using Benjamini-Hochberg procedure. For the comparative analysis of the two groups, Wald test is used in DESeq2 to identify differentially expressed taxa. The p-values obtained from Wald test were then adjusted for multiple testing using the Benjamini-Hochberg procedure to Control FDR. Significant taxa (*p* < 0.05) identified by DESeq2 were visualized using volcano and bar plots.

### Evaluation of the effect of treatment on gut and brain

To further investigate the molecular implications of antibiotic treatment and FQ-35 supplementation, we performed histopathological analysis to assess intestinal tissue integrity and evaluated the expression of key gut integrity and neuroinflammatory markers across the study groups.

#### Histopathology analysis

Intestinal tissues were fixed in formalin (10%) in phosphate buffered saline (PBS). The tissues were subsequently embedded in paraffin, sectioned and proceeded for haematoxylin-eosin (H & E) staining. The stained sections were observed under digital microscopy and checked for morphological and histological changes.

#### Gut integrity and neuroinflammatory markers

The frozen gut samples were used to study the expression of the gut integrity markers. Total RNA from the tissues was isolated using Takara RNAisoplus reagent (Takara, Japan). cDNA was synthesized using the kit method (Biorad, California, USA) and qRT-PCR was carried out using iTaq (Univer SYBR Green Supermix, Biorad, California, USA) on a Biorad CFX Opus real-time system following manufacturer’s instructions. The expression of markers for gut integrity (Zonulin, ZO1 and Occludin) and neuroinflammation (TNFa, TLR and IL-1B) were quantified. KiCqStart™ primer sequences—R_il1b_2, R_Tnf_1, and R_Ocln_1—pre-validated by the manufacturer (Sigma-Aldrich, USA), were used for the expression analysis of IL-1β, TNF-α, and Occludin, respectively. Primer sequences for ZO-1, TLR-4, and Zonulin were selected based on previously published study^82^. The specificity of each primer pair was reconfirmed through melt-curve analysis, and sequence information is provided in Table 1. The qPCR reaction conditions involved an initial denaturation at 95 °C for 35 s followed by extension at 60 °C for 30 s for 39 cycles. The relative gene expression was calculated using 2^−∆∆CT^ method.

**Table 1:** Details of primer sequences used for qRT-PCR amplification of gut integrity and neuroinflammatory markers.

Marker	Forward Primer	Reverse Primer
ZO1	5’-ATGGTTGGTATGGTGCCCTG-3’	5’-TTGTAGCACCATCCGCCTTC-3’
Zonulin	5’-ACTGGGTCCA GGAAACAATG-3’	5’-TCCTCTTCCAGGGTGAATTG-3’
Occludin	5’-CTAGCTGATGTAATCTATGGC-3’	5’-CTGAGAAGGGTTA TGTTTTCAC-3’
TLR-4	5’-ATCTGAGCTTCAACCCC CTG-3’	5’-TGTCTCAATTTCACACACCTGGAT-3’
TNFa	5’-CTCACACTCAGATCATCTTC-3’	5’-GAGAACCTGGGAGTAGATAAG-3’
IL-1B	5’-AAGAATCTATACCTGTCCTGTG-3’	5’-CAAACTCCACTTTGGTCTTG-3’

#### Estimation of acetylcholinesterase 

Acetylcholinesterase (AchE) activities in the gut and brain samples were analysed according to the method of Ellman^83^. Tissue samples were homogenised in PBS, total protein estimated using Bradford method^83^. AchE activities were determined in a 200 µL reaction mixture made up of 72 µL 1mM DTNB (Ellman’s reagent (5,5′-dithiobis-2-nitrobenzoic acid)) sodium bicarbonate (1.5 mg/mL) added during the preparation of DTNB, 71 µL PBS, 25 µL sample, 32 µL 1mM ATCH (acetylthiocholine iodide). Corrected blank absorbance calculated from blank 1 (72 µL 1mM DTNB, 96µL 1x PBS, and 32 µL 1mM ATCH) and blank 2 (25 µL Sample, 143 µL PBS, and 32 µL ATCH). The enzyme activities were quantified at 412 nm. One unit of AchE activity was defined as the amount of enzyme required to convert 1 µmol of acetylcholine iodide per minutes per mg protein under standard conditions.

### Statistical analysis

Statistical analysis was performed using GraphPad Prism version 9.0 (GraphPad Software, San Diego, CA). Data normality was assessed using the Shapiro-Wilk test prior to conducting parametric analyses. Outliers were identified using the built-in GraphPad outlier test (ROUT method) with a Q value of 1%. One-way ANOVA followed by Dunnett’s post hoc test was performed. Results are expressed as mean ± SD with *p < 0.05, **p < 0.01, ***p < 0.001 indicating statistical significance.

## Supplementary Information

Below is the link to the electronic supplementary material.

Supplementary Material 1

Supplementary Material 2

Supplementary Material 3

Supplementary Material 4

## Data Availability

The metagenome data supporting the study’s findings are deposited at the NCBI Sequence Read Archive (SRA) under BioProject ID PRJNA1202064 and the data supporting the findings of this study are available from the corresponding author upon reasonable request.
