# Supplementary material for: Quercetin hybrid-hydrogel microparticles modulate gut microbiota and improve memory in an antibiotic-induced dysbiosis rat model
Source: Sci Rep. 2025 Nov 25;15:42429. doi: 10.1038/s41598-025-26608-7 (PMC12661057; doi:10.1038/s41598-025-26608-7)
Supplement: Supplementary file 4 — Supplementary Material 4 [file 41598_2025_26608_MOESM4_ESM.docx]

**Quercetin Hybrid-Hydrogel Microparticles Modulate Gut Microbiota and Improve Memory in an Antibiotic-Induced Dysbiosis Rat Model**

**Supplementary Figure File**

1. **Day15**


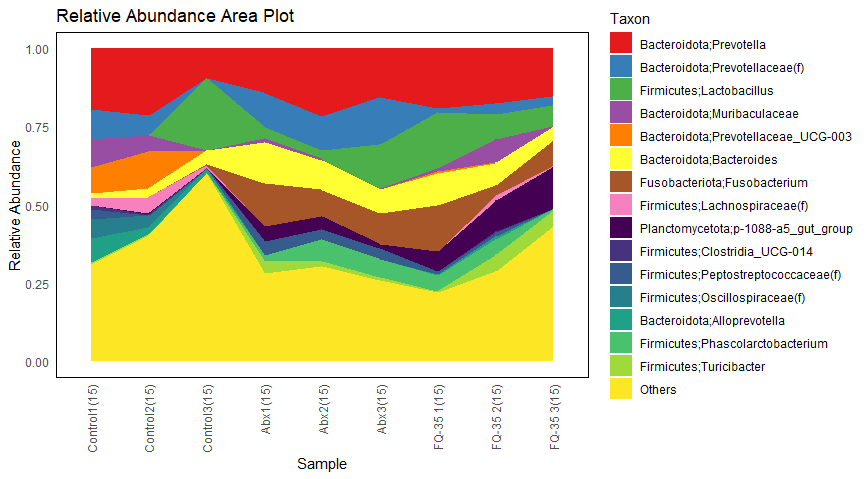


1. **Day30**


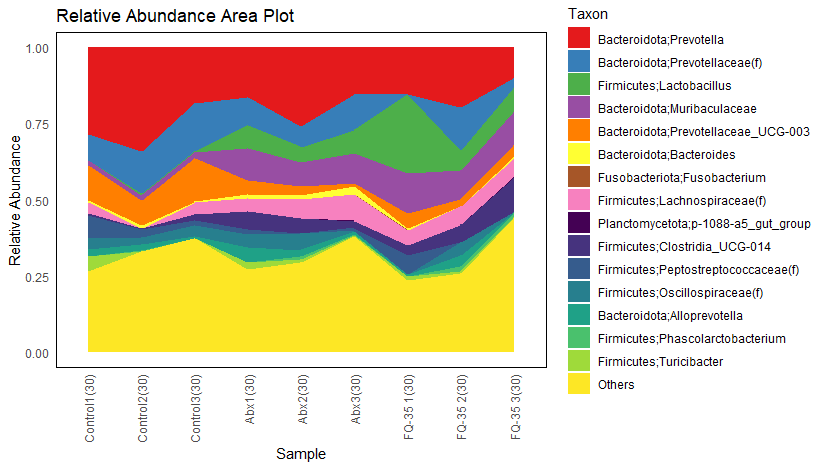


**Supplementary Fig1.** Relative abundance of the top fifteen microbial Genera distribution across Control, antibiotic (Abx) and Quercetin (FQ-35) groups **(A)** post-antibiotic treatment and **(B)** post-intervention treatment.


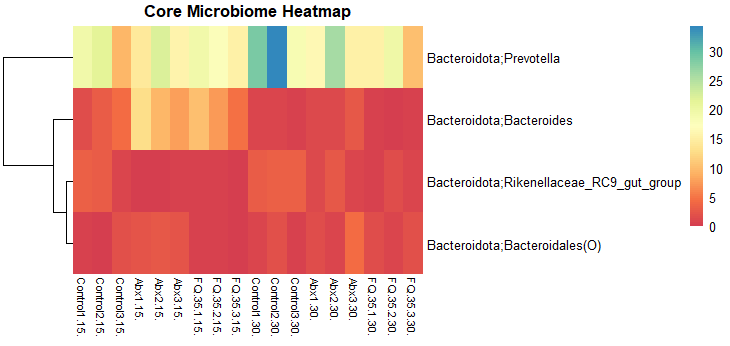


**Supplementary Fig2.** Core microbiome from post antibiotic and post intervention treatment. The heatmap displays four genera of core microbiome distribution across the groups at the two time points.


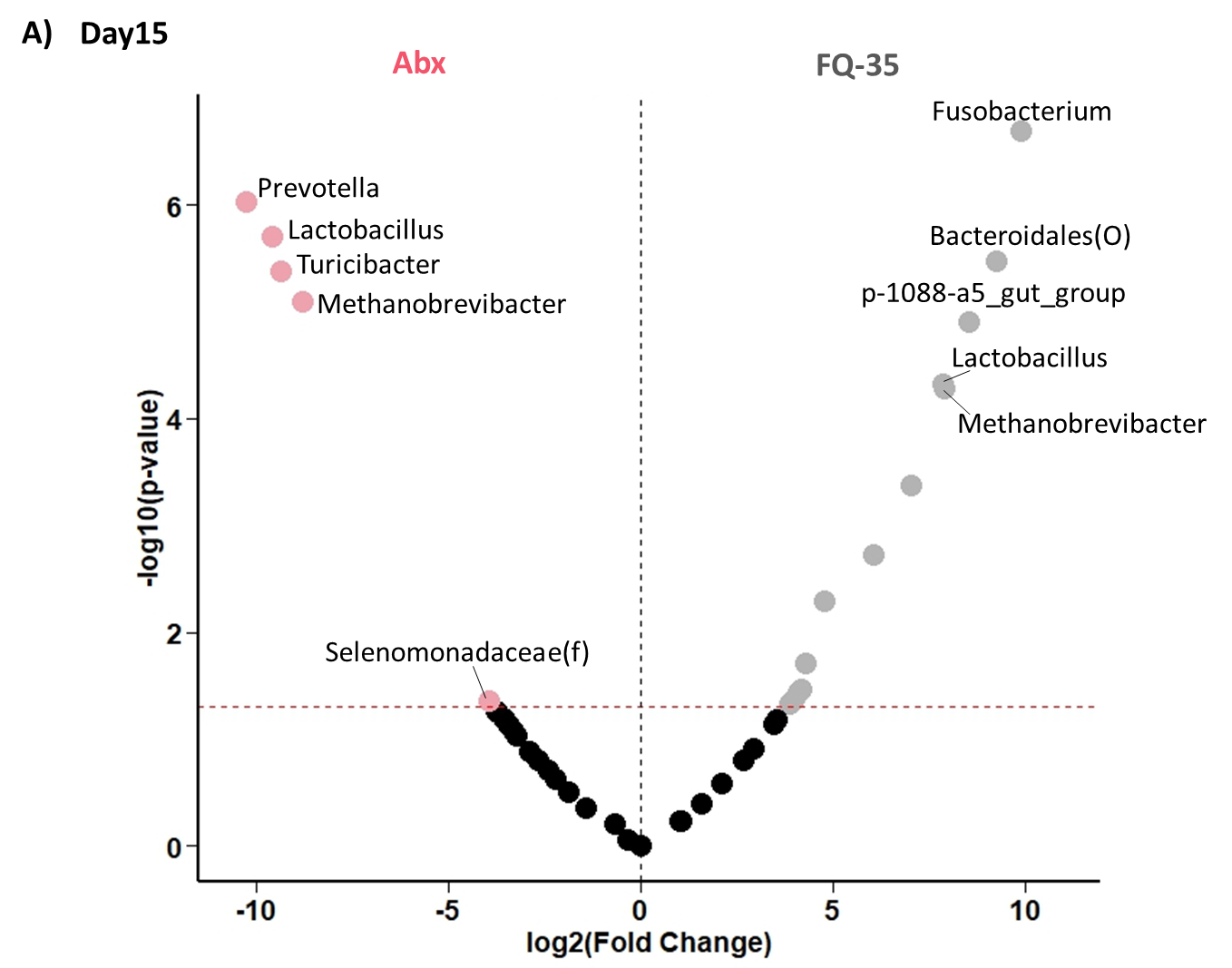


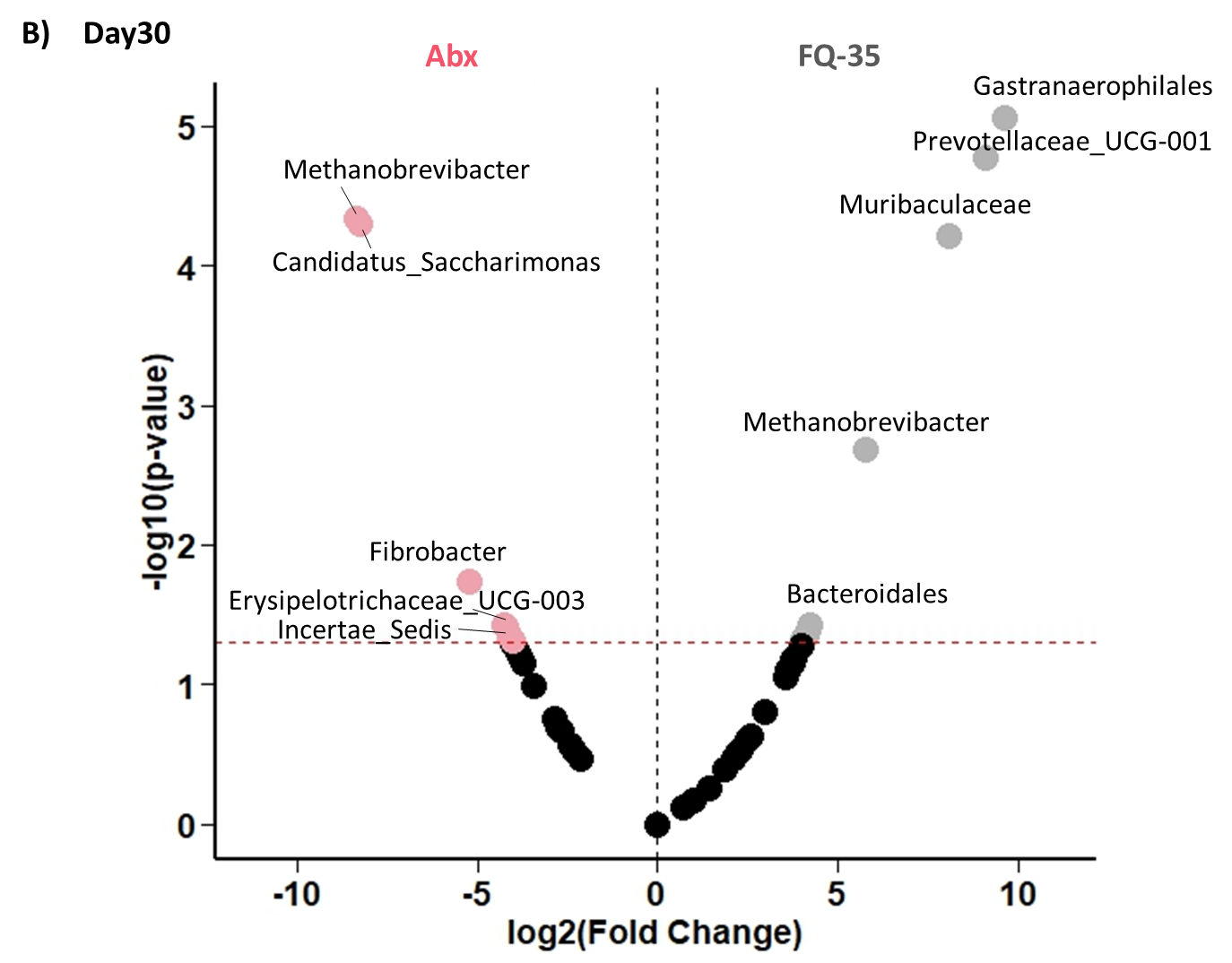


**Supplementary Fig3.** Differential analysis of bacterial genera is obtained from DESeq2. The volcano plot showing **(A)** significant taxa associated with a comparison between Abx and FQ-35 group observed on post-antibiotic treatment. **(B)** Significant taxa associated with a comparison between Abx and FQ-35 group observed on post-intervention treatment. The top five genera with Log2 FC >1 and p-value < 0.05 is represented respectively in both time points.
